# Community-Based Participatory Approaches to Improve Diet Quality and Food Security in Rural Appalachia: Outcomes from the Laurel HARVEST Study

**DOI:** 10.3390/nu18172813

**Published:** 2026-08-27

**Authors:** Makenzie Barr-Porter, Courtney Luecking, Stacey Slone, Dawn Brewer, Joley Helton, Ashton Potter, Kathryn Cardarelli

**Affiliations:** 1 Department of Dietetics and Human Nutrition, University of Kentucky, Lexington, KY 40546, USA; courtney.luecking@uky.edu (C.L.); dawn.brewer@uky.edu (D.B.); joley.helton@uky.edu (J.H.); 2 Dr. Bing Zhang Department of Statistics, University of Kentucky, Lexington, KY 40536, USA; stacey.slone@uky.edu; 3 The Food Connection, University of Kentucky, Lexington, KY 40506, USA; ashtonpotter@uky.edu; 4 Department of Epidemiology and Population Health, University of Louisville, Louisville, KY 40202, USA; kathryn.cardarelli@louisville.edu

**Keywords:** food security, diet quality, nutrition education, rural, CBPR, PSE, healthy eating index, carotenoids

## Abstract

**Background:** Rural Appalachian communities experience high rates of food insecurity, poor diet quality, and chronic disease. Community-based participatory research (CBPR) and policy, systems, and environmental (PSE) approaches may improve nutrition-related outcomes through locally tailored interventions. This study evaluated the impact of Laurel HARVEST (Helping Appalachia Restore a Vibrant Food Environment for Self-Sufficiency, Together) on dietary quality and food security in Appalachian Kentucky. **Methods:** A prospective cohort study was conducted in Laurel County, Kentucky (intervention) and Pike County, Kentucky (comparison). Adults aged ≥18 years were recruited at baseline (2022–2023) and followed through 2025. CBPR-guided interventions included nutrition education, recipe card dissemination, raised bed gardens, and community mini-grants. Outcomes included Healthy Eating Index (HEI) scores derived from 24 h dietary recalls, dermal carotenoid concentrations measured using the Veggie Meter^®^, and food security assessed with the USDA 10-item Household Food Security Module. **Results:** A total of 282 participants were enrolled at baseline (Laurel n = 121; Pike n = 161), and 147 completed follow-up assessments. Dermal carotenoid scores increased significantly more in Laurel County (37.3 points) than in Pike County (13.3 points) (*p* = 0.032). No significant between-county differences were observed for total HEI score or food security. **Conclusions:** CBPR-driven, multilevel interventions were associated with modest improvements in fruit and vegetable intake but did not significantly improve overall dietary quality or food security. Longer-term structural and policy-level approaches may be needed to address nutrition and food access challenges in rural Appalachian communities.

## 1. Introduction

Generally, rural populations experience disproportionately greater health disparities and higher burdens of chronic disease compared with urban populations. Rates of cardiovascular disease, stroke, and diabetes are consistently higher among rural residents than among urban counterparts [1]. Many social determinants of health (SDOH) contribute to these disparities, including higher rates of poverty in rural areas, lower educational attainment, transportation challenges, environmental factors, and food insecurity [1]. Kentucky, specifically, ranks among the top ten states most affected by lifestyle-related chronic conditions closely related to geography, culture, and economic challenges that are common within the Appalachian region (423 counties across 13 states that span the Appalachian Mountain range of the United States) [2,3]. Appalachian counties in Kentucky have slightly higher percentages of obesity and diabetes than those of non-Appalachian counties and significantly more deaths due to heart disease, cancer, stroke, and diabetes [4].

Among the SDOH, food insecurity in the United States is a financial and lifestyle stressor. Rural households (16.1%) experience food insecurity at higher rates than urban households (14.1%) [5]. Geographic isolation further exacerbates vulnerability in rural regions, where limited transportation, lower wages, underemployment, and higher poverty rates contribute to increased risk [5]. Kentucky is among the top five states with the highest prevalence of food insecurity [6] with its Appalachian counties experiencing higher rates than non-Appalachian equivalents [7]. Food insecurity is often associated with poor diet quality related to economic hardship [7]. In rural areas, nutrient-dense foods, such as fruits and vegetables, can be difficult to access in food environments with few healthy food vendors. This encourages the consumption of highly processed, low nutrient-dense foods. Consistent with these challenges, only approximately 9% of Kentucky residents meet recommended daily fruit and vegetable intake guidelines [8].

Culture in rural areas is one of kinship and community trust. The utilization of current community resources such as Cooperative Extension services, food pantries, and religious organizations is beneficial to build trust among community members. Community-based participatory research (CBPR) is an approach that equitably involves community members, researchers, and other contributors in the research process [9]. The goal is to create a positive community-wide impact that is sustainable and long-lasting. Cooperative Extension personnel represent a strong, trusted partner when planning interventions within rural or isolated communities. Extension agents are embedded within communities and collaborate directly with residents to deliver education, technical assistance, and outreach programming [10].

Policy, systems, and environmental (PSE) interventions at multiple levels of the socioecological model offer promising strategies in improving food insecurity and food access, which, in turn, can ultimately improve chronic disease [11]. The current program, Laurel HARVEST (Helping Appalachia Restore a Vibrant Food Environment for Self-Sufficiency, Together), aimed to identify whether utilizing PSE approaches, coupled with CBPR methodology, improves dietary quality and food security status among rural adults.

## 2. Materials and Methods

Laurel County was chosen as the intervention county for this study. Laurel County is situated in the southeastern part of Appalachian Kentucky, with 61% of the county’s population living in a low-population-density area [12]. Poverty is higher in Laurel County (19.5%) compared to the entirety of the state (16.5%) [13]. Likewise, food insecurity rates are higher in the county (18.8%) than in Kentucky as a whole (16.6%) [14]. Laurel County is affected by chronic diseases, with rates of adult obesity (41%) and type 2 diabetes (13%) higher than the United States national average (38% with obesity; 11% with diabetes) [12,13,15,16].

A 2025 Community Health Implementation Strategy and Plan (CHISP) from CHI St. Joseph Hospital in London, KY (Laurel County), identified “nutrition, physical activity, and weight” as one of the significant community health needs, along with diabetes, heart disease, and stroke [16]. One of the anticipated impacts of this CHISP, under the umbrella of “nutrition, physical activity, and weight”, included “reduced food insecurity, increased access and consumption of healthy foods”, parallel with the goals of the Laurel HARVEST project. This shows community buy-in and identification of the same goals for the county.

Assets within the county related to food security and healthy eating include farmers markets, Cooperative Extension programs, and nutrition education for school-aged youth [13]. A 2023 assessment by the University of Kentucky’s Cooperative Extension Service for Laurel County confirmed priorities, emphasizing the need to address chronic disease and increase access to safe, affordable, fresh foods, and support for local agriculture [17]. Respondents highlighted under the category of Food Access, Nutrition and Healthy Eating, “practical education and support on topics related to hunger and food insecurity; food banks and pantries; nutrition education and access to healthy food; education on growing, preserving, and canning food.” Respondents also stressed enhancing support for local agriculture such as, “practical education and support for small and family farms and new farmers; support for organic (or sustainable) and low chemical farming; training on farmland preservation; training on farm safety and food safety on farms, and farmers markets; market support for locally produced foods; education on off-grid living and home gardening; etc.” [17].

Pike County, Kentucky, was used as the comparison county in this study. Pike County, which is also designated Appalachian, is located 120 miles east of Laurel County and has similar sociodemographic characteristics. In Pike County, 84.8% of the population lives in a low-population-density area [18]. The prevalence of poverty is at 22.4% and remains higher than the state’s total percentage [19]. Food insecurity rates are similar to Laurel County at 22.5% [20]. Pike County is affected by chronic diseases, with 41% of residents having obesity [18] and 13% reporting diabetes [18]. The geographic distance between the counties ensured no spillover effects of the interventions.

### 2.1. Laurel HARVEST Development Phase

As CBPR was the main framework of the overarching concept of the Laurel HARVEST project, a Community Advisory Board (CAB) was established within the county to include community champions, organizations, and leaders within the space of nutrition and food security. Further details of the CAB development and methods have been previously described [21]. The Laurel County CAB assisted in the decisions surrounding the needs of their community along with intervention ideas. Throughout the working relationship with the CAB, intervention ideas were generated surrounding the main goals of the project: improving dietary quality and food security. Suggested CAB intervention ideas were explored by the research team for feasibility, and approximately 50% of the ideas came to fruition (explained further in Luecking et al. [21]).

Throughout the program lifespan, individual, interpersonal, and community-level efforts of interventions were implemented. As explained in our previous work describing the CAB process [21], the members identified target groups of individuals to include working families, relatives raising children, and lower-income households. After understanding the current landscape of the county related to ongoing food and nutrition initiatives, main intervention ideas were formulated between the CAB and the research team. Intervention ideas were placed into overarching themes of nutrition education and food systems (Table 1).

#### 2.1.1. Nutrition Education

**Figure 1:**
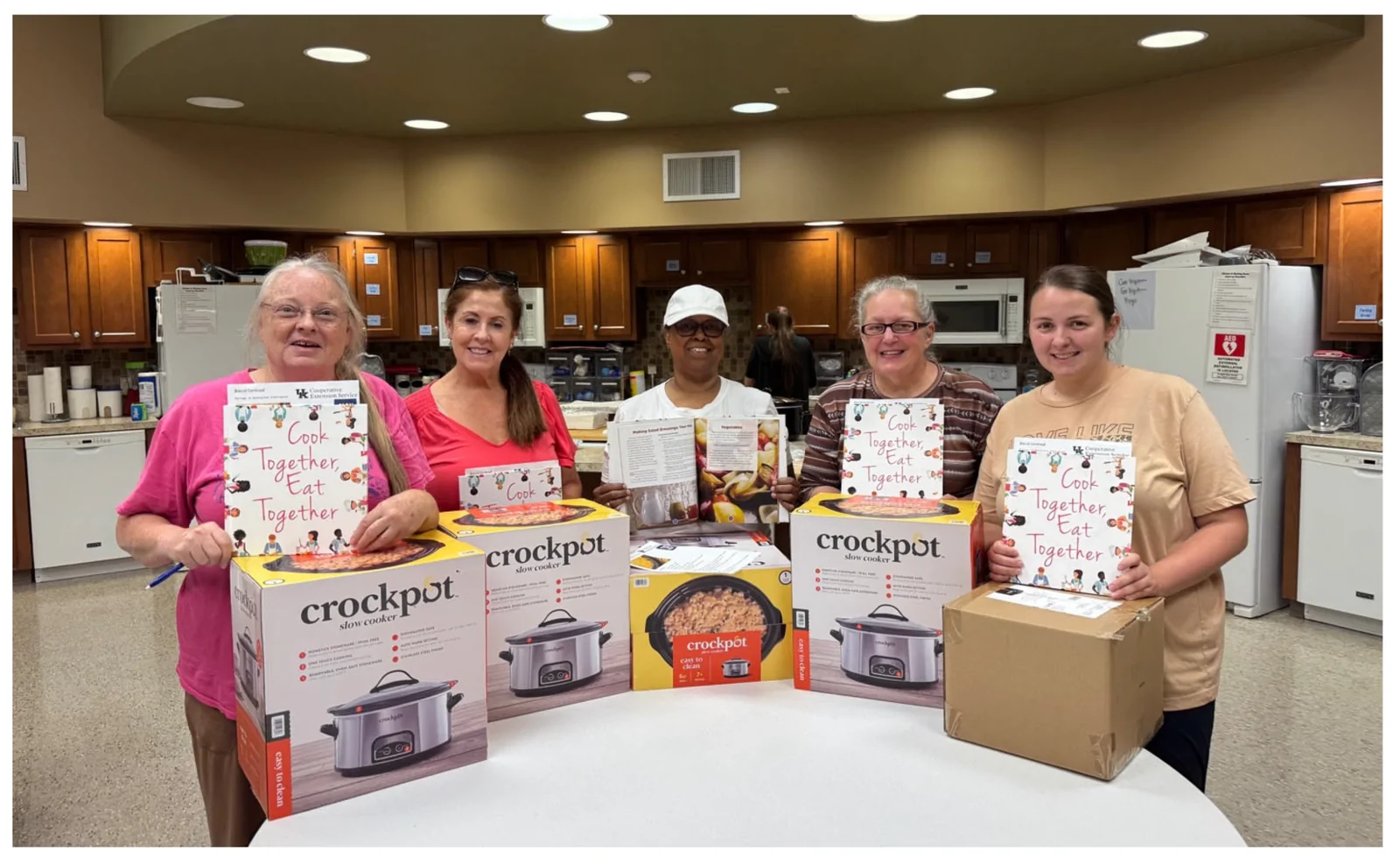
Cook Together, Eat Together.

The CAB chose Cook Together, Eat Together (CTET) (also described in a forthcoming article) as a primary nutrition education intervention. CTET is a 10-session evidence-based program developed by the University of Kentucky’s Cooperative Extension to provide “cooking socials” for families to practice cooking skills together [22]. The CTET was originally developed for a low-income audience to provide simple, budget-friendly meals to improve dietary quality. This program was chosen by the CAB to be offered to the community, with the aim of engaging the target populations, though it was identified that the population they are trying to reach may not have the capacity to attend 10 in-person sessions in the afternoon/evening hours of the day. To overcome this challenge, the CAB recommended the program be shortened and adapted into a hybrid version (i.e., some of the sessions offered live, online). With the collaboration of the Laurel County Extension Family and Consumer Sciences agent and Nutrition Education Program assistant, the program was tailored to offer a hybrid version. In the adapted version, 6 sessions were offered with 2 in-person and 4 virtual sessions. Content included social interaction with the group, nutrition and/or food resource management skill education (i.e., grocery list building, reusing leftovers, knife skills), and preparing two recipes. This program was offered eight times between May 2023 and June 2025 and had 46 total research participants (Figure 1).

**Figure 2:**
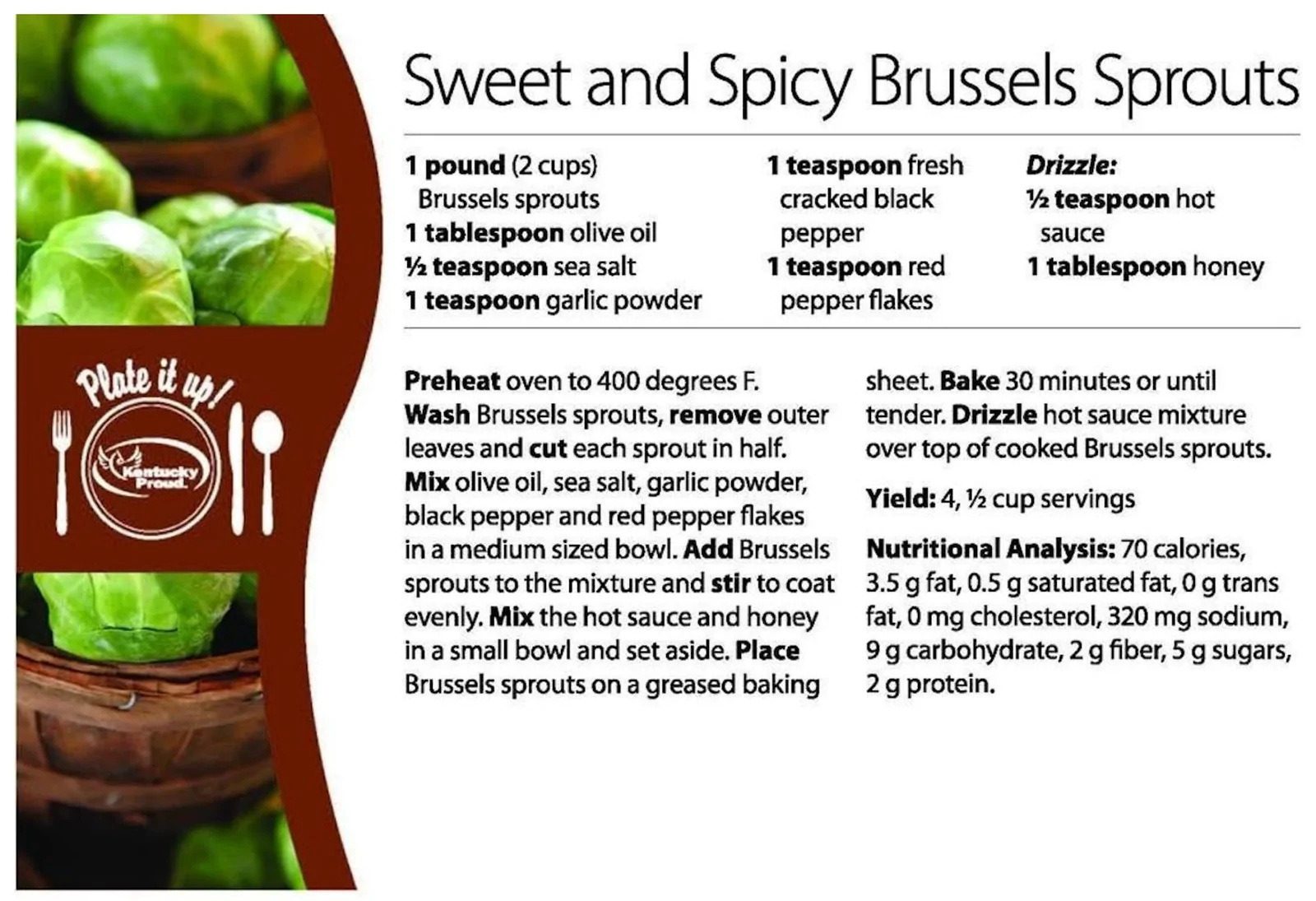
Plate it up! Kentucky Proud Recipe Card.

To create additional connections between county residents and the nutrition education content from the county Extension Office, the CAB recommended recipe cards be shared where feasible. This intermittent intervention was established during CAB meetings and included utilizing the recipe cards at the local food pantries. An example included “Plate it up!” recipe cards. Plate it Up! cards are developed and owned by the University of Kentucky Cooperative Extension Service and were designed to provide options for healthy recipes that utilize local Kentucky Proud food products. This effort, chosen by the CAB, was aimed at improving dietary quality through healthy food items that are easier to find locally and by providing these recipe cards at a food pantry with items they receive and may not know how to incorporate into a recipe. These recipe cards (https://fcs.mgcafe.uky.edu/plate-it-up, accessed on 20 July 2026; Figure 2) were printed at postcard size and were made available at various locations such as the Cooperative Extension Office, at food banks and food pantries, and on Facebook. These efforts were low-effort and ongoing through the project years 2023–2026.

#### 2.1.2. Food Systems

One idea that was formulated due to an existing partnership with the health department was to introduce, or re-introduce, raised bed gardens throughout the community to improve food access and dietary quality for those who may experience food insecurity. With help from local farmers, gardeners, and Horticulture Extension staff on the CAB, partnerships were formed across the county to identify locations interested in establishing raised bed gardens. Partners also helped to identify the capacity and staff to maintain the gardens. Within the county, three local childcare facilities (e.g., Head Start, childcare facility for parents in recovery, and church-based childcare) and a local low-income housing complex expressed interest in the program and developed opportunities with members of their organization to ensure sustainability of the garden. For example, gardening education and maintenance were built into day-to-day activities with daycare children (i.e., allowing children to water plants and pull weeds).

**Figure 3:**
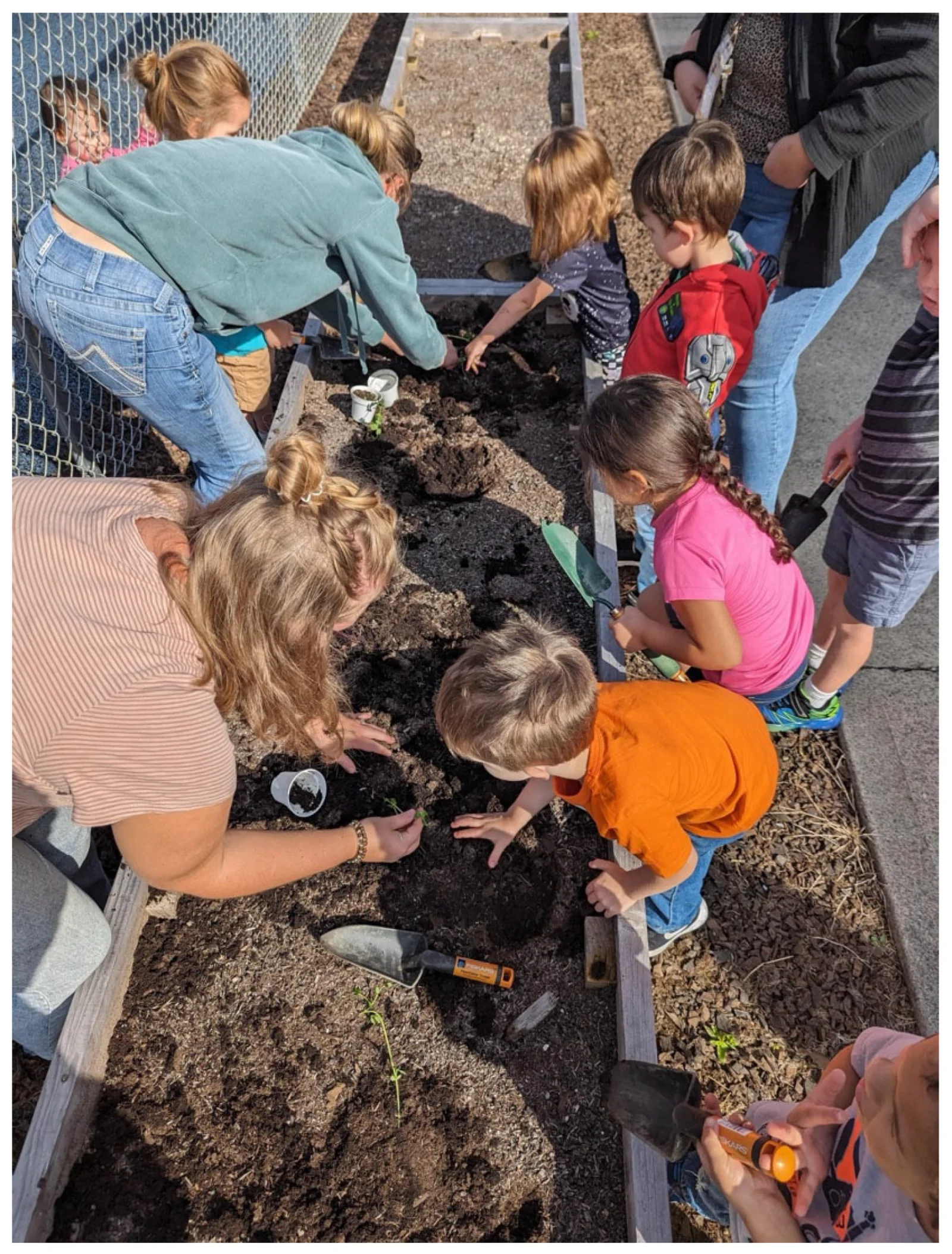
Raised bed gardens at childcare facility.

During the summer of 2024, gardens were planted at 3 childcare facilities (Figure 3); raised bed gardens were planted by research staff, a horticulture Extension agent and assistants, volunteers, and help from the facility staff and students. Crops planted included cherry tomatoes, tomatoes, bell peppers, carrots, radishes, cucumbers, green beans, and summer squash.

To ensure further community buy-in and support, planting was accomplished in partnership with the Master Gardeners program at the Cooperative Extension office. The Master Gardeners program is a training certification offered through Cooperative Extension in which enrollees attend a series of classes providing basic horticulture training in several topics (i.e., Volunteerism, Botany, Soils, Pathology, Entomology, and Pesticides), take a certification exam, and continue to maintain volunteer hours throughout the year. For Laurel County Master Gardener participants, the raised beds at the low-income housing complex were offered as an option for receiving volunteer hours to weed, re-soil, plant, and provide basic education. Periodically, Master Gardener volunteers would tend to the garden beds and assist the staff with any garden maintenance.

#### 2.1.3. Mini Grants

A core component of the Laurel HARVEST project aimed to support self-sufficiency within the community to address food insecurity and fruit and vegetable consumption. In the spirit of CBPR, the research team offered mini-grant funds to individuals and organizations in Laurel County to address one of the main objectives within their own work. Two separate calls for mini-grant proposals were made in January 2025 and November 2025.

Mini-grant eligibility criteria required projects to focus on the Laurel County population, demonstrate broad community support (including specific plans for involving community members as members of the target population, local health-related organizations, and/or local businesses), run between 6 and 12 months, and meet one of the Laurel HARVEST main objectives (1. Increase fruit and vegetable consumption or 2. Reduce food insecurity) and demonstrate sustainability after mini-grant funding ended. Applicants completed an online application packet that included a cover sheet, background and statement of need, proposed project with objectives and timeline, budget, and budget justification. Funding was limited to materials and supplies for project activities only and could not be used for salary or labor, clinical services, phone bills, rent, utilities, computer software, catered food, or alcohol.

In total, 6 mini grants were funded, including 4 at USD 3000 and 2 at USD 4500. For recipients, required activities during the project period included evaluation of funding impact (i.e., what the money was used for, how many people were reached, number of meals provided, number of class sessions held), a written final report describing accomplishments, and attending regular meetings with the HARVEST research project manager.

Mini-grant recipients were invited to present their work at a CAB meeting and completed a mid-point and final grant report. Mini-grant recipients utilized the funds for the purchase of a mobile cooking cart, infrastructure for a greenhouse, summer feeding program for children and families, food resource boxes for individuals with diabetes, development of food pantries at Head Start facilities, and ‘Harvest Hope’ food boxes for resource-scarce individuals.

### 2.2. Research Phase

The study was conducted in accordance with the Declaration of Helenski, and the protocol received expedited review and approval at the University of Kentucky’s Institutional Review Board (#64602). Alongside intervention implementation, we conducted a prospective cohort study to evaluate whether community-level implementation of Laurel HARVEST was associated with changes in food security and dietary behaviors. To assess intervention effects, adults from Laurel County (intervention community) and Pike County (comparison community) were recruited and followed over time. Because the intervention was intended to reach the broader community, cohort enrollment was independent of participation in Laurel HARVEST programming. Baseline data were collected between 2022 and 2023, prior to widespread implementation of intervention activities, and follow-up assessments were conducted in 2025. Given the intervention’s organic approach and broad community reach, evaluation focused on changes among community residents rather than outcomes among specific intervention program participants alone.

#### 2.2.1. Cohort Participant Recruitment and Eligibility

Cohort study eligibility criteria included adults aged 18 years and older who live in Laurel and/or Pike Counties, are not currently pregnant, speak and understand English, and report no plans to move within 3 years. Interested individuals completed an online screener for eligibility and once meeting the inclusion criteria, an enrollment session was scheduled.

#### 2.2.2. Intervention Community: Laurel County, KY

To identify prospective cohort participants in the intervention county, the CAB, and local Cooperative Extension professionals leveraged their established networks for sharing recruitment information. Participants were recruited from various locations within the county, including the Women, Infants, and Children (WIC) program office at the Laurel County Health Department, Cooperative Extension office and social media, and the Come-Unity Cooperative Care food pantry. Recruitment began in December 2022, and data collection took place between December 2022 through November 2023.

#### 2.2.3. Control Community: Pike County, KY

In Pike County, the research team leveraged their Family and Consumer Sciences Extension Agent, who has deep relationships in the community, to recruit participants. Recruitment began in January 2023 and data collection took place between April 2023 through October 2023. Control participants were largely (>90%) recruited from clientele of a local community health initiative that provide no-cost support for connecting individuals with local medical, social, and environmental services at no cost to the client. Within the Pike County, arts-based programming was offered as the control condition.

### 2.3. Data Collection

Primary outcomes for the Laurel HARVEST program were (1) healthy eating and (2) food security. To determine changes on an individual level, we employed reliable and valid quantitative assessment measures (described below). At scheduled baseline appointments, participants arrived at the county Cooperative Extension office (both Laurel & Pike) and were consented into the study. A trained research coordinator performed all data collection efforts in intervention and control counties. Research personnel completed a dermal carotenoid scan. Surveys were administered in-person on tablet devices through the REDCap platform 17.4.1 [23,24], and dietary recall was administered through the Automated Self-Administered 24 h recall from the National Cancer Institute (ASA-24). Surveys were verbally administered to participants needing assistance with operating the tablet or completing the survey. Following data collection, participants received a gift card for their time (USD 30 at baseline, USD 45 at follow-up). The same instruments and procedures were used in both the intervention and comparison counties in years 1 and 4 (baseline and follow-up).

Survey demographics asked age, birth sex, years of residence in county, racial identity, ethnicity, current relationship, highest education completed, annual household income, tobacco use, and food assistance use.

#### 2.3.1. Dietary Quality

Dietary quality of participants was assessed through multiple measures, including dietary recall, validated survey tools, and dermal carotenoid scan.

Participants completed the Automated Self-Administered 24 h recall from the National Cancer Institute (ASA-24) [25]. ASA-24 was used to calculate the Healthy Eating Index (HEI) score at baseline and for follow-up [26]. The HEI measurements capture changes in the thirteen dietary components outlined in the Dietary Guidelines for Americans 2020–2025 [27]. A maximum score—5 or 10 points—can be reached for each dietary component, depending on the component. Points are awarded when the amounts consumed meet the standard for a particular dietary component. Amounts that do not meet the standard receive fewer points, with zero being the minimum score, and the maximum total HEI score is 100. Dietary components that yield higher scores with greater intake include total fruits, total vegetables, total protein foods, seafood and plant protein, dairy, whole grains, oils, whole fruits, and greens and legumes. Dietary components that yield higher scores with lower consumption include saturated fat, sodium, added sugars, and refined grains. Data were analyzed to assess changes in total HEI scores and subscores including fruit and vegetable consumption.

The Expanded Food and Nutrition Education Program Food and Physical Activity Questionnaire (EFNEP-FPAQ) [28] is a validated tool used to evaluate self-reported behavior change among individuals participating in the USDA’s EFNEP program, which is a low-income audience. The most recent version of the FPAQ includes 30 questions to collect self-reported behaviors pertaining to five domains: diet, physical activity, food safety, food security, and food resource management.

As an additional measure of healthy eating, we used the VEGGIE METER™ (Longevity Link Corp., Salt Lake City, UT, USA) [29,30,31] to measure dermal carotenoids using non-invasive RS Spectroscopy. Carotenoids are a biological marker of fruit and vegetable consumption. This non-invasive measurement of dermal carotenoids has been validated against the standard of serum carotenoids in adults [32,33,34,35]. Scoring for the Veggie Meter ranges from 0 to 800 with a higher score indicating a diet with higher fruit and vegetable intake. As carotenoids are stored in the skin, they serve as a better marker of usual fruit and vegetable ingestion compared to serum carotenoids, which fluctuate with daily dietary intake. The dermal carotenoid measurement reflects fruit and vegetable intake over approximately 2 to 4 weeks, typically taking 4 to 6 weeks of consistent carotenoid-food intake to have a measured change [33,34]. To obtain the measurement, subjects placed their pointer finger into the machine for 20 s while their finger is squeezed. Three replicates were taken per person at 20 s per replicate [35]. Average of scores were used for analysis.

#### 2.3.2. Food Security

The USDA’s 10-item household food security screener module was used to assess food security status [36]. Example questions include “The food that (I/we) bought just didn’t last, and (I/we) didn’t have money to get more.” and “(I/we) couldn’t afford to eat balanced meals.” Answer choices included often, sometimes, never, and don’t know. The number of affirmative answers (sometimes or often) from each of the 10 questions were counted for a total score of 0–10 with higher scores indicating more food insecurity. For those indicating having children in the household, an additional 8 questions were asked to report food insecurity impacting children.

#### 2.3.3. Power and Sample Size Justification

While the study was originally designed for data collection at three time points, the study team decided to eliminate the interim time point due to the interruption with the COVID-19 pandemic. Assuming an intraclass correlation of 0.65, 107 participants per county provided 80% power to see a percent change of 7.3% in the total HEI. To allow for 30% attrition, 160 participants per county was the recruitment goal. The recruitment goal was met in Pike County with 161 participants enrolled; however, Laurel County’s recruitment was lower with just over 75% of the goal recruited (n = 121).

#### 2.3.4. Statistical Methods

Baseline characteristics are summarized using medians and interquartile ranges (IQRs) for continuous measures and counts and percentages for categorical measures. Differences between participants in Laurel compared to those in Pike County and participants who completed the follow-up visit compared to those who were lost to follow-up were analyzed using Kruskal–Wallis tests (continuous measures) and Chi-square or Fishers Exact tests (categorical measures), as appropriate.

Normality was assessed in the outcomes visually and with the Shapiro–Wilkes tests. The HEI total scores and food security scores were relatively normal. The changes in the scores over time were assessed with a repeated measures linear model with a compound symmetry correlation structure. The model adjusted for birth sex and age at baseline and the growing season during data collection (to address in-season produce and subsequent consumption), tobacco use (Yes/No/Former) and food assistance (Yes/No) at each visit. A significant difference in the change in the scores was determined from the county by visit interaction.

While the dermal carotenoid and kilocalorie outcomes were highly skewed at the individual time points, the change in the outcomes (follow-up–baseline) was relatively normal and met assumptions of the ANOVA. For consistency, the ANCOVA models mimicked the repeated measures models as closely as possible controlling for birth sex, age, growing season, tobacco use (Yes/No/Former) and food assistance (Yes/No) at baseline. One extreme outlier was removed from the dermal carotenoid models. Models including the outlier produced similar results. Statistical significance was set a priori at 0.05. All statistical analyses were completed using SAS 9.4 (SAS Institute, Inc., Cary, NC, USA).

## 3. Results

At baseline (Table 2), participants in Laurel (intervention) and Pike (control) counties had some differences among birth sex, relationship status, employment status, and type of food assistance use (all *p* < 0.05). There was no significant difference between intervention and control counties among food security status (*p* = 0.069).

Significant differences were detected between counties at baseline for FPAQ responses and HEI scores. Among the FPAQ dietary quality questions, the only significant difference at baseline was the number of times per day participants consumed vegetables (*p* = 0.032).

Differences in baseline HEI values included component scores for energy in kcals (*p* = 0.022), total vegetables (*p* = 0.004), greens and beans (*p* = 0.036), whole fruit (*p* = 0.025), dairy (*p* = 0.036), and seafood and plant protein (*p* = 0.036), and total score (*p* = 0.004), with Laurel County participants having a higher HEI total score (48.8 vs. 42.9) (Appendix A.2, Table A2).

Veggie Meter results (dermal carotenoids) were not significantly different at baseline between the two counties, with Laurel County participants beginning at 146 points and Pike County participants at 137 points (*p* = 0.07).

For food security status, participants mostly screened as “high” food security status in both Laurel County (42.0%) and Pike County (28.6%).

At follow-up (Table A1), 70 Laurel County participants and 77 Pike County participants attended data collection (52.1% retention). Characteristics of participants attending and not attending the follow-up data collection were compared (Appendix A.1; Table A1). Participants attending the follow-up visit were older in age (*p* = 0.001) and had differences among food insecurity status (*p* = 0.032). Among those attending follow-up data collection, 38.8% were categorized as “high food security status” (n = 57), followed by 25.2% “low food security status” (n = 37). For those lost to follow-up, the highest percentage was categorized as “very low food security status” (30.1%; n = 40), followed by “high food security status” (29.3%; n = 39).

To examine changes in EFNEP-FPAQ dietary quality from baseline to post-program, participants’ changes in responses were tracked. Participant self-report indicated changes in dietary behaviors from baseline to post-program. Table 3 indicates change or maintenance in behaviors. All behaviors predominantly remained the same across counties. Variables with a higher percentage of individuals that “increased” included red/orange vegetables in both counties, dark green vegetables in the intervention county, and beans and peas in the control county. There were no significant differences in percent change in behavior between counties.

The dermal carotenoids (veggie meter) of the Laurel County participants increased on average 37.3 (95%CI: 17.6, 56.9) points, while Pike County participants saw a smaller average increase of 13.3 (95%CI: −6.1, 32.7) points (*p* = 0.032), which indicates the change in fruit and vegetable intake in Laurel County participants was almost triple that of Pike County. The only other significant covariate in the model was tobacco use (*p* = 0.037) (Table 4). On average, while participants in both counties lowered their caloric intake over the course of the study, neither decrease was significant. In addition, the changes were not significantly different between counties. The only significant factor in the model was tobacco use (*p* = 0.006).

Total HEI score decreased slightly for Laurel County participants (−3.7%, 95%CI: −8.1, 0.8), while Pike County participants remained the same (0.1%, 95%CI: −4.0, 4.2). Overall, Laurel County participants decreased their total HEI score by 3.8% (95%CI: −9.6, 2.1) more than the Pike County participants (*p* = 0.203). Birth sex and baseline age were also significant in the model (*p* = 0.05 and 0.02, respectively). Similarly, the Food Security score remained the same on average for the Laurel County participants (0.03, 95%CI: −0.73, 0.78) and decreased for the Pike County participants (−0.67, 95%CI: −1.39, 0.05). Laurel County participants raised their Food Security scores by 0.70 points more than the Pike County participants (95%CI: −0.30, 1.69).

## 4. Discussion

The current study aimed to evaluate the impact of a community-based participatory research (CBPR) approach to multilevel policy, systems, and environmental (PSE) interventions in improving dietary quality and food security status among rural Kentucky adults. Supported through community partnerships such as the Community Advisory Boards (CABs) and implemented with and through Cooperative Extension, the Laurel HARVEST program produced mixed findings. Notably, participants in the intervention county (Laurel County, KY) demonstrated significant increases in dermal carotenoid scores compared to the control county (Pike County, KY), suggesting an improvement in fruit and vegetable intake. Likewise, increases in dietary quality on EFNEP-FPAQ included red/orange vegetables in both counties, dark green vegetables in the intervention county, and beans and peas in the control county. However, there were no significant differences in overall dietary quality through the HEI or in food security status.

The observed increase in dermal carotenoids, despite minimal change in HEI scores, suggests nuanced improvements in fruit and vegetable intake that may not be fully captured by composite dietary indices. Dermal carotenoids, measured via the Veggie Meter, provide an objective assessment of fruit and vegetable intake, whereas the HEI tends to reflect self-reported daily intake [29,30,33,35,37]. When utilizing the HEI in this study with rural adults, participants were self-reporting one day of intake (i.e., the previous 24 h) at each data collection time point. While the Veggie Meter score reflects 2–4 weeks of fruit and vegetable intake, the HEI score was indicative of daily, self-reported intake across two data collection days. At baseline, several HEI component scores were higher among Laurel County residents compared to Pike County (Appendix A.2; Table A2). The addition of the Veggie Meter scores at baseline reflects low dietary diversity of fruits and vegetables compared with existing literature of Veggie Meter scores among adults averaging approximately 200–350 [30,38]. With baseline HEI scores in both counties meeting less than 50% of DGA recommendations, at follow-up, we saw HEI scores decreased by about 4% for Laurel residents, while remaining consistent in Pike County. HEI component scores were examined following the program, and there were no significant differences between counties. Although participants confirmed that data were taken from a typical day of eating, scores are still taken from single days and self-reported. With inadequate HEI scores based on DGA recommendations, it is clear there remains a need to improve dietary quality among our vulnerable residents.

Within the larger cohort outcomes, there was an absence of significant improvements in food security, highlighting the complexity of addressing structural determinants of food access in rural communities. While the intervention included some downstream approaches to food security such as funding mini-grants supporting food distribution, upstream solutions are needed. Food security is shaped by social determinants, including employment, income, transportation, and the food system [39,40]. As the Laurel HARVEST program was relatively short in duration, the needed scale of change may not have been sufficient to address these factors. These findings underscore the importance of implementation timelines when evaluating community-based PSE programs. Changes in county-level dietary quality and food security often occur over extended periods of time with substantial partnership and tailoring to the community content. Though there is an absence of significant changes in our main outcome measures of diet quality and food insecurity, findings should be considered in the context of the evolving nature of community interventions, and that meaningful individual-level changes may emerge only after sustained exposure and broader community adoption.

The CBPR framework was a strength of the current study and ensured interventions were community-supported and tailored to the priorities established by the CAB. As previously published, the research team fostered meaningful relationships with the CAB throughout the duration of the program to help guide and inform selection and adaptations of interventions to fit local context [21]. The CBPR approach aligns with best practices for conducting lifestyle and community programming, particularly in rural areas. Community buy-in, trust, knowledge, and ownership are important to implementing research in isolated communities and creating sustainable change. Through an iterative process with the CAB, we were able to collaborate on several intervention ideas. Although the CAB developed strong ideas within the food systems sector of the program, barriers arose that limited the scale of implementation. For example, efforts to establish a collaboration with the local farmers market or expand into a mobile market were hindered by transportation and staffing. With this lack of multiple ideas coming to fruition, the dosage of the intervention components within the county was ultimately reduced, which can impact potential effects.

Though some ideas were unable to be implemented, we had open lines of communication with our CAB that included emails, monthly or bimonthly meetings, and phone calls with our program coordinator. These “touch points” allowed for a more personal touch and availability for us to work through challenges or pivot from ideas that were at an impasse. This work highlights the importance of strong partnership and community engagement, and also structural and resource availability when implementing PSE strategies.

Overall, diet and food security are difficult to achieve and sustain due to a variety of internal and external factors of daily life. Though the study produced limited, small findings, this is consistent with literature demonstrating that multilevel, community-based interventions can produce modest improvements in dietary behaviors in rural populations [41,42,43]. Behavior change interventions are important across the social ecological model framework but also need to be supported with structural change to make lasting improvements to health. In conjunction with programming from the current study such as hybrid cooking socials, implementation of raised bed gardens, and mini-grant projects, mechanisms to enhance long-term impact may include strengthening of food systems and infrastructure supports. Likewise, the continuation of CBPR approaches and partnership with Cooperative Extension can offer the foundation for delivering such interventions in rural settings.

### Limitations

Several limitations should be considered when interpreting these findings. Although recruitment goals were met in the comparison county, only approximately 75% of the target sample was recruited in the intervention county, and overall retention at follow-up was 52.1%. At baseline, recruitment and data collection happened quickly for the control county due to collaborators who had a strong network of clientele who were interested in participation. In the intervention county, though partnerships existed, the window of recruitment was widened with dwindling interest despite continued efforts (e.g., making new connections in the county, various modality uses for connecting with potential participants, and utilizing our network to reach out directly to their clientele). To keep recruitment in the same seasonality of the state (i.e., changes in resident willingness for activity during winter months) and because intervention components needed to begin, baseline data collection was completed at 75%. For follow-up data collection retention, there was approximately 2 years between baseline and follow-up data collection, which may result in a decline in participant retention. Having a mid-point data collection at 1 year may have resulted in stronger follow-up. This reduced sample size may have limited the ability to detect statistically significant differences in outcomes, particularly for measures such as HEI and food security. We assessed demographic differences among participants who completed follow-up assessments; participants were older in age and in the “high” category of food security status for Laurel County (Appendix A.1; Table A1). Follow-up data points from those experiencing lower food insecurity status were missing from our intervention county and may represent cohort findings from a more stable population or less food-insecure subgroup, making observed intervention effects less noticeable.

Although validated measurement tools were used for the current study, limitations remain. Self-reported dietary intake is a challenge within community nutrition research. Difficulty with individual participant recall, along with potential social desirability bias, complicates outcomes in this work. Dietary changes within the population may not have been captured through the HEI due to sensitivity, along with the tool being used for only one day of recall at baseline and post-program. While we utilized the Veggie Meter as an objective measure to overcome some of the challenges with dietary recall data, it does not fully capture broader behaviors. Finally, the study sample was predominantly white, older, and rural, leading to limited generalizability to a more diverse population in a different geographical setting.

Additionally, intervention dosage was unable to be captured due to the nature of the study being county-wide and a relatively short duration for change. Likewise, some aspects were passive in nature, such as recipe card distribution. With this variability, it is difficult to discern whether observed or unobserved changes were due to intervention type and dosage. With this structure, individuals in the main cohort may not have been uniformly exposed to the intervention elements such as CTET, gardens, or mini grant reach. Interventions were chosen by the CAB to target their chosen population of low-income, working families, though enrollment in the programs was not restricted, rather a community-wide invitation. Although some programs (i.e., raised bed gardens) were implemented specifically in childcare facilities that only enroll lower-income families, others, such as CTET, are required to be open to the community at large through the Cooperative Extension Office. To ensure due diligence in reaching our target population, conscious efforts to recruit for programs at locations in the county such as food pantries and WIC offices. Future iterations of similar programs may consider more targeted recruitment and engagement strategies as well as evaluating individual-level exposure in each intervention type to assess if those participating are receiving the intended benefit.

With the CBPR nature of the project, the CAB suggested several intervention components that were unable to materialize. Many strategies were explored by the research team and CAB to understand viability or pressure points. Though ideas were continuously adapted and addressed, this lack of certain ideas being implemented may indicate a gap in the intervention that is needed within the community. As the CAB selected programming with the knowledge of their community and population in mind, this inability to implement some programs may have reduced overall effectiveness. However, after moving past “non-starts,” the CAB identified tangential programming to address that sector in a different mechanism (i.e., food systems shift from a mobile farmers market to raised garden beds).

While the intervention programs within Laurel HARVEST were able to be successfully implemented, lack of significant findings may be attributed to the length of the program. Our programs within this PSE model may require longer exposure within a large setting such as a county-level to see measurable impact. Though PSE interventions were not developed, this may be due to project-related constraints (i.e., community readiness or grant ending) rather than lack of feasibility. The timeframe needed for PSE intervention work is a particularly important consideration, especially in rural or geographically isolated communities. Limited food system infrastructure, different food acquisition practices, transportation barriers, general lack of time challenges among rural residents, and an overall lack of trust in outside entities (i.e., university researchers) can impact intervention uptake among working families. Additional longitudinal research is needed within the realm of rural PSE interventions to provide appropriate length of programming and adequate intensity to produce sustained change in dietary quality and food security.

An additional challenge of the CAB was the lack of members directly representing the target populations of interest. The CAB identified importance with low-income and/or working families, as well as multi-generational families. Unfortunately, though we had groups and organizations represented that serve these target populations, we lacked representation from those groups directly to ensure feasibility of intervention components. This absence of target population representation on the CAB may be due to the aforementioned lack of trust in outside intervention or general constraints of time beyond their personal responsibilities. Future work should aim to ensure continual integration and collaboration with members of target populations.

Finally, as this project aimed to encompass the breadth of Policy, Systems, and Environmental (PSE) approaches, based on CAB priorities and resources available in the county, policy-level intervention components were not implemented. As these structural changes are time- and resource-heavy, buy-in from the community is imperative to maintain sustainability beyond the confines of grant-related funding. However, a common challenge in CBPR and CABs is that members often balance multiple responsibilities across their professional, personal, and community roles. Importantly, many CAB members are already highly engaged leaders within their communities, routinely volunteering their time and assuming additional responsibilities to support fellow community members and local initiatives. As a result, these individuals often have limited capacity to take on new commitments, making it difficult to ask them to contribute further time and effort to policy-related activities that require substantial and sustained engagement. Creating efficient and feasible ways to integrate project work into CAB members’ current roles may be beneficial in creating reasonable added obligations.

## 5. Conclusions

In conclusion, this study demonstrates that CBPR-driven, multilevel interventions can yield modest improvements in fruit and vegetable consumption in rural communities, though broader impacts on dietary quality and food security may require longer-term and more intensive structural changes. Though the program had attenuated or modest effects on the targeted outcomes, the work remains valuable when addressing health in rural populations. Utilization of pairing community-engaged approaches with investments in rural food systems and infrastructure can achieve meaningful advances in nutritional health. However, lasting improvements in diet and food security require upstream solutions (i.e., policy change). Future work may benefit from a focus on capturing broader nutritional intake data, continuing strong partnership collaborations, strengthening food systems strategies, and improving retention of participants and reach programming.

## Figures and Tables

**Table 1:** HARVEST intervention descriptions.

Intervention	Program Description	Target Audience	Time Period
Cook Together, Eat Together	Six-session, hybrid format, family-focused nutrition education curriculum teaching cooking, nutrition, and budgeting skills.	Families with youth, caregivers, seniors, and adults	May 2023–June 2025
Recipe Cards	Recipe cards were printed and supplied to the regional food bank distribution center in Laurel County and made available to patrons of partner food pantries. Additionally, recipe cards were supplied to the local Cooperative Extension office and shared online via social media (e.g., Facebook).	Resource-scarce individuals and community members served through Cooperative Extension.	July 2023
Raised Bed Gardens	Raised garden beds were installed at three local childcare facilities and one low-income housing complex. Crops included seasonal vegetables that could easily be harvested and incorporated into meals.	Youth and resource-scarce individuals.	Summer 2024
Mini-grants	Community organizations received funding for six projects to promote fresh fruit and vegetable consumption and reduce food insecurity in Laurel County. Funded initiatives included a summer feeding program, food resource boxes for diabetes, food boxes for resource-scarce individuals, development of food pantries at Head Start facilities, a mobile cooking cart education kit, and infrastructure for a greenhouse for education and providing produce.	Varied by program	January 2025–October 2026

**Table 2:** Baseline demographics.

		Baseline		
Variable	Category	Laurel	Pike	*p*-Value
n		121	161	
Age		61 (38.5, 68.0)	56 (47.0–66.0)	0.381
Birth sex	Male	16 (13.3%)	51 (31.7%)	<0.001 *
	Female	104 (86.7%)	110 (68.3%)	
Race	American Indian/Alaska Native	0 (0.0%)	1 (0.6%)	0.642
	Black or African American	1 (0.8%)	1 (0.6%)	
	White	117 (96.7%)	155 (97.5%)	
	More than one race	2 (1.7%)	0 (0.0%)	
	Other	1 (0.8%)	1 (0.6%)	
Ethnicity	Not Hispanic or Latinx	108 (94.7%)	147 (94.8%)	1.000
	Hispanic or Latinx	2 (1.8%)	3 (1.9%)	
	Decline to answer	4 (3.5%)	5 (3.2%)	
Current relationship status	Single (never married)	10 (8.5%)	28 (17.6%)	0.018 *
	Married/domestic partnership	51 (43.2%)	76 (47.8%)	
	Widowed	23 (19.5%)	23 (14.5%)	
	Divorced	32 (27.1%)	25 (15.7%)	
	Separated	1 (0.8%)	6 (3.8%)	
	Other	1 (0.8%)	1 (0.6%)	
Highest education completed	8th grade or less	8 (7.0%)	16 (10.5%)	0.082
	9th–11th grade	7 (6.1%)	21 (13.7%)	
	High school diploma/GED	37 (32.2%)	54 (35.3%)	
	Some College	26 (22.6%)	27 (17.6%)	
	Associate’s degree or Vocational	14 (12.2%)	18 (11.8%)	
	Bachelor’s degree	14 (12.2%)	14 (9.2%)	
	Advanced degree	9 (7.8%)	3 (2.0%)	
Employment status	Employed full-time	22 (18.5%)	28 (17.6%)	<0.001 *
	Employed part-time	9 (7.6%)	6 (3.8%)	
	Unemployed by choice	4 (3.4%)	7 (4.4%)	
	On sick leave or disability	9 (7.6%)	33 (20.8%)	
	A homemaker	12 (10.1%)	18 (11.3%)	
	Retired due to illness	24 (20.2%)	47 (29.6%)	
	Retired, not due to illness	32 (26.9%)	15 (9.4%)	
	Unemployed, laid off	7 (5.9%)	5 (3.1%)	
Current annual household income	Less than USD 20,000	45 (37.8%)	78 (48.8%)	0.156
	USD 20,000–29,999	25 (21.0%)	35 (21.9%)	
	USD 30,000–39,999	11 (9.2%)	12 (7.5%)	
	USD 40,000–49,999	7 (5.9%)	13 (8.1%)	
	USD 50,000–59,999	10 (8.4%)	10 (6.3%)	
	USD 60,000 or greater	16 (13.4%)	8 (5.0%)	
	Decline to answer	5 (4.2%)	4 (2.5%)	
Tobacco user	No	82 (71.3%)	93 (60.4%)	0.178
	Yes (or stopped within the last year)	21 (18.3%)	39 (25.3%)	
	Former tobacco user	12 (10.4%)	22 (14.3%)	
Food assistance use	WIC	6 (5.0%)	9 (5.6%)	0.815
	SNAP or food stamps	41 (33.9%)	58 (36.0%)	0.709
	Food pantry	48 (39.7%)	35 (21.7%)	<0.001 *
	Friends and family	9 (7.4%)	22 (13.7%)	0.098
	Free school meals	15 (12.4%)	7 (4.3%)	0.013 *
	Other	6 (5.0%)	7 (4.3%)	0.809
	None	45 (37.2%)	68 (42.2%)	0.392
USDA Household Food Security Screener	High	50 (42.0%)	46 (28.6%)	0.069
	Marginal	24 (20.2%)	30 (18.6%)	
	Low	24 (20.2%)	43 (26.7%)	
	Very Low	21 (17.6%)	42 (26.1%)	
HEI Total Score	(score 0–100%)	48.8 (40.0–56.2)	42.9 (34.2–53.0)	0.004 *
Veggie Meter	(score 0–800)	146.0 (113.0–178.0)	137.0 (97.0–171.0)	0.066

* *p* < 0.05; GED = General Education Development; WIC = Women, Infants and Children; SNAP = Supplemental Nutrition Assistance Program; USDA = United States Department of Agriculture; HEI = Healthy Eating Index.

**Table 3:** EFNEP-FPAQ dietary quality survey response changes by county.

FPAQ Variable	County	Decreased	Stayed Same	Increased
Eat fruit (Times/Day)	Intervention	18 (25.7%)	32 (45.7%) ^	20 (28.6%)
	Control	23 (30.3%)	34 (44.7%) ^	19 (25.0%)
Eat vegetables (Times/Day)	Intervention	19 (27.9%)	25 (36.8%) ^	24 (35.3%)
	Control	21 (28.0%)	38 (50.7%) ^	16 (21.3%)
Different vegetables (Kinds/Day)	Intervention	14 (20.6%)	31 (45.6%) ^	23 (33.8%)
	Control	19 (25.3%)	29 (38.7%) ^	27 (36.0%)
Last 7 days, # red/orange vegetables	Intervention	25 (35.7%)	17 (24.3%)	28 (40.0%) ^
	Control	26 (33.8%)	22 (28.6%)	29 (37.7%) ^
Last 7 days, # dark green vegetables	Intervention	19 (27.5%)	21 (30.4%)	29 (42.0%) ^
	Control	27 (35.5%)	29 (38.2%) ^	20 (26.3%)
Last 7 days, # beans and peas	Intervention	25 (36.2%) ^	25 (36.2%) ^	19 (27.5%)
	Control	19 (24.7%)	27 (35.1%)	31 (40.3%) ^

^ indicates the highest percentage for each variable ranking, per county.

**Table 4:** Main outcomes.

Outcome Change (Follow-Up–Baseline)	Laurel County (Intervention)		Pike County (Control)		Difference (Laurel Co–Pike Co)	
	Mean (95%CI)	*p*-Value	Mean (95%CI)	*p*-Value	Mean (95%CI)	*p*-Value
Carotenoids	37.3 (17.6, 56.9)	0.0003 *	13.3 (−6.1, 32.7)	0.18	24.0 (2.1, 45.9)	0.032 *
Caloric Intake	−1.7 (−374.7, 371.2)	0.99	−115.6 (−495.5, 264.3)	0.55	113.8 (−297.2, 524.8)	0.58
HEI Total Score	−3.7 (−8.1, 0.8)	0.11	0.13 (−4.0, 4.2)	0.95	−3.8 (−9.6, 2.1)	0.20
Food Security	0.03 (−0.73, 0.78)	0.94	−0.67 (−1.39, 0.05)	0.07	0.70 (−0.30, 1.69)	0.17

Carotenoid score in points (0–800); caloric intake in calories; HEI score in percentage (0–100%); food security score in points (0–10). CI = Confidence Interval. Mean changes and CI estimated from the multivariate models. * *p* < 0.05.

## Data Availability

The data presented in this study are available on request from the corresponding author due to ongoing data analyses and publishing.

## Appendix A

### Appendix A.1

**Table A1:** Follow-up participant descriptive statistics.

		Attrition		
Variable	Category	Follow-Up	No Follow-Up	*p*-Value
n		147	135	
Age		62 (50.0–68.0)	54 (38.5–64.0)	0.001 *
Birth sex	Male	31 (21.1%)	36 (26.9%)	0.256
	Female	116 (78.9%)	98 (73.1%)	
Race	American Indian/Alaska Native	0 (0.0%)	1 (0.7%)	0.415
	Black or African American	2 (1.4%)	0 (0.0%)	
	White	141 (96.6%)	131 (97.8%)	
	More than one race	2 (1.4%)	0 (0.0%)	
	Other	1 (0.7%)	1 (0.7%)	
	Decline to answer	0 (0.0%)	1 (0.7%)	
Ethnicity	Not Hispanic or Latinx	126 (93.3%)	129 (96.3%)	0.633
	Hispanic or Latinx	3 (2.2%)	2 (1.5%)	
	Decline to answer	6 (4.4%)	3 (2.2%)	
Current relationship status	Single (never married)	13 (9.2%)	25 (18.5%)	0.246
	Married/domestic partnership	66 (46.5%)	61 (45.2%)	
	Widowed	26 (18.3%)	20 (14.8%)	
	Divorced	33 (23.2%)	24 (17.8%)	
	Separated	3 (2.1%)	4 (3.0%)	
	Other	1 (0.7%)	1 (0.7%)	
Highest education completed	8th grade or less	10 (7.2%)	14 (10.8%)	0.505
	9th–11th grade	12 (8.7%)	16 (12.3%)	
	High school diploma/GED	49 (35.5%)	42 (32.3%)	
	Some College	26 (18.8%)	27 (20.8%)	
	Associate’s degree or Vocational	15 (10.9%)	17 (13.1%)	
	Bachelor’s degree	18 (13.0%)	10 (7.7%)	
	Advanced degree	8 (5.8%)	4 (3.1%)	
Employment status	Employed full-time	23 (16.0%)	27 (20.1%)	0.041 *
	Employed part-time	9 (6.3%)	6 (4.5%)	
	Unemployed by choice	4 (2.8%)	7 (5.2%)	
	On sick leave or disability	16 (11.1%)	26 (19.4%)	
	A homemaker	20 (13.9%)	10 (7.5%)	
	Retired due to illness	36 (25.0%)	35 (26.1%)	
	Retired, not due to illness	32 (22.2%)	15 (11.2%)	
	Unemployed, laid off	4 (2.8%)	8 (6.0%)	
Current annual household income	Less than USD 20,000	61 (42.4%)	62 (45.9%)	0.321
	USD 20,000–29,999	32 (22.2%)	28 (20.7%)	
	USD 30,000–39,999	13 (9.0%)	10 (7.4%)	
	USD 40,000–49,999	6 (4.2%)	14 (10.4%)	
	USD 50,000–59,999	13 (9.0%)	7 (5.2%)	
	USD 60,000 or greater	15 (10.4%)	9 (6.7%)	
	Decline to answer	4 (2.8%)	5 (3.7%)	
Tobacco user	No	97 (69.8%)	78 (60.0%)	0.229
	Yes (or stopped within the last year)	26 (18.7%)	34 (26.2%)	
	Former tobacco user	16 (11.5%)	18 (13.8%)	
Food assistance use	WIC	10 (6.8%)	5 (3.7%)	0.246
	SNAP or food stamps	43 (29.3%)	56 (41.5%)	0.032
	Food pantry	39 (26.5%)	44 (32.6%)	0.256
	Friends and family	13 (8.8%)	18 (13.3%)	0.229
	Free school meals	13 (8.8%)	9 (6.7%)	0.496
	Other	7 (4.8%)	6 (4.4%)	0.899
	None	64 (43.5%)	49 (36.3%)	0.215
USDA Household Food Security Screener	High	57 (38.8%)	39 (29.3%)	0.032 *
	Marginal	30 (20.4%)	24 (18.0%)	
	Low	37 (25.2%)	30 (22.6%)	
	Very Low	23 (15.6%)	40 (30.1%)	
HEI Total Score	(score 0–100%)	47.3 (38.9–56.0)	42.7 (36.4–53.6)	0.074
Veggie Meter	(score 0–800)	147.0 (106.0–185.0)	137.0 (100.0–167.0)	0.125

* *p* < 0.05.

### Appendix A.2

**Table A2:** HEI Component Scores Baseline and Post.

	Baseline			Post	
	Laurel	Pike	*p*-Value	Laurel	Pike
N	121	161		70	77
Energy (kcal)	1554.3 (1187.8–2112.2)	1318.5 (829.8–1970.5)	0.022 *	1465.7 (1030.1–2094.4)	1462.4 (967.8–2047.6)
Total Vegetables	4.0 (2.0–5.0)	2.9 (0.8–5.0)	0.004 *	4.1 (2.0–5.0)	3.2 (0.9–5.0)
Greens and Beans	0.0 (0.0–4.5)	0.0 (0.0–0.0)	0.036 *	0.0 (0.0–4.3)	0.0 (0.0–0.5)
Total Fruit	0.4 (0.0–4.6)	0.0 (0.0–3.1)	0.066	0.3 (0.0–4.2)	0.0 (0.0–3.5)
Whole Fruit	0.5 (0.0–5.0)	0.0 (0.0–3.9)	0.025 *	0.5 (0.0–5.0)	0.0 (0.0–4.7)
Whole Grains	0.0 (0.0–4.0)	0.0 (0.0–1.5)	0.066	0.0 (0.0–5.6)	0.0 (0.0–1.9)
Dairy	5.7 (2.2–9.4)	3.7 (1.1–8.1)	0.036 *	5.8 (1.2–9.8)	3.7 (1.4–9.3)
Total Protein Foods	5.0 (4.1–5.0)	5.0 (3.3–5.0)	0.323	5.0 (4.3–5.0)	5.0 (3.2–5.0)
Seafood and Plant Proteins	0.3 (0.0–5.0)	0.0 (0.0–3.3)	0.036 *	1.4 (0.0–5.0)	0.0 (0.0–5.0)
Fatty Acid Ratio	4.1 (0.7–7.4)	4.7 (1.7–8.0)	0.313	4.4 (0.7–9.2)	4.7 (1.5–8.2)
Sodium	3.7 (0.5–6.5)	3.1 (0.0–7.6)	0.373	4.4 (0.3–7.0)	2.5 (0.0–5.9)
Refined Grains	6.8 (3.2–10.0)	5.7 (2.0–10.0)	0.237	6.5 (3.2–10.0)	5.7 (2.7–9.9)
Saturated Fats	3.9 (0.4–7.1)	5.0 (1.2–8.3)	0.138	4.1 (0.3–8.2)	5.4 (1.4–8.3)
Added Sugar	8.9 (4.8–10.0)	8.0 (3.8–10.0)	0.219	9.8 (5.5–10.0)	7.4 (4.0–10.0)
TOTAL HEI-2015 SCORE	48.8 (40.0–56.2)	42.9 (34.2–53.0)	0.004 *	49.9 (41.5–57.5)	44.4 (34.7–54.5)

* *p* < 0.05.
